# A Novel GATA1 Variant in the C-Terminal Zinc Finger Compared with the Platelet Phenotype of Patients with A Likely Pathogenic Variant in the N-Terminal Zinc Finger

**DOI:** 10.3390/cells11203223

**Published:** 2022-10-14

**Authors:** José M. Bastida, Stefano Malvestiti, Doris Boeckelmann, Verónica Palma-Barqueros, Mira Wolter, María L. Lozano, Hannah Glonnegger, Rocío Benito, Carlo Zaninetti, Felix Sobotta, Freimut H. Schilling, Neil V. Morgan, Kathleen Freson, José Rivera, Barbara Zieger

**Affiliations:** 1 Departmento de Hematología, Complejo Asistencial Universitario de Salamanca (CAUSA), Instituto de Investigación Biomédica de Salamanca (IBSAL), Universidad de Salamanca (USAL), 37007 Salamanca, Spain; 2 Department of Pediatrics and Adolescent Medicine, Division of Pediatric Hematology and Oncology, Faculty of Medicine, Medical Center–University of Freiburg, 79106 Freiburg, Germany; 3 Servicio de Hematología y Oncología Médica, Hospital Universitario Morales Meseguer, Centro Regional de Hemodonación, Universidad de Murcia, IMIB-Pascual Parrilla, CIBERER-U765, 30003 Murcia, Spain; 4 Instituto de Investigación Biomédica de Salamanca (IBSAL), Instituto de Biología Molecular y Cellular del Cáncer (IBMCC), Centro de Investigación del Cáncer (CIC), Universidad de Salamanca-Consejo Superior de Investigaciones Cientificas (CSIC), 37007 Salamanca, Spain; 5 Institut für Transfusionsmedizin, Universitätsmedizin Greifswald, 17475 Greisfwald, Germany; 6 Children’s Hospital, Kantonsspital Luzern, 6000 Lucerne, Switzerland; 7 Institute of Cardiovascular Sciences, College of Medical and Dental Sciences, University of Birmingham, Birmingham B15 2TT, UK; 8 Department of Cardiovascular Sciences, Center for Molecular and Vascular Biology, KU Leuven, 3000 Leuven, Belgium

**Keywords:** platelet pathophysiology, inherited platelet defects, bleeding, GATA1

## Abstract

The GATA1 transcription factor is essential for normal erythropoiesis and megakaryocytic differentiation. Germline GATA1 pathogenic variants in the N-terminal zinc finger (N-ZF) are typically associated with X-linked thrombocytopenia, platelet dysfunction, and dyserythropoietic anemia. A few variants in the C-terminal ZF (C-ZF) domain are described with normal platelet count but altered platelet function as the main characteristic. Independently performed molecular genetic analysis identified a *novel* hemizygous variant (c.865C>T, p.H289Y) in the C-ZF region of GATA1 in a German patient and in a Spanish patient. We characterized the bleeding and platelet phenotype of these patients and compared these findings with the parameters of two German siblings carrying the likely pathogenic variant p.D218N in the GATA1 N-ZF domain. The main difference was profound thrombocytopenia in the brothers carrying the p.D218N variant compared to a normal platelet count in patients carrying the p.H289Y variant; only the Spanish patient occasionally developed mild thrombocytopenia. A functional platelet defect affecting αIIbβ3 integrin activation and α-granule secretion was present in all patients. Additionally, mild anemia, anisocytosis, and poikilocytosis were observed in the patients with the C-ZF variant. Our data support the concept that GATA1 variants located in the different ZF regions can lead to clinically diverse manifestations.

## 1. Introduction

Inherited platelet disorders (IPDs) account for a great proportion of hemorrhagic diatheses. Historically, they are classified either as quantitative or as qualitative platelet defects according to whether the major clinical feature is thrombocytopenia or impaired platelet function [1,2,3]. Over the past decade, however, genetics has unveiled a new horizon to better characterize and understand the etiology of IPDs. Several transcription factor defects have been identified in patients affected by IPDs. The variants occur in genes coding for transcription factors regarded as crucial for the physiologic development of hematopoietic cells, most prominently RUNX1, ETV6, FLI1, GFI1B, and GATA1 [4]. They form dimers with partner transcription factors. Depending on the combining partner, they bind to different promotor and repressor regions and finally regulate lineage-defining gene expression. This is probably the reason why transcription factor defects generally affect multiple cell lineages [5,6]. 

In particular, the transcription factor GATA1 is highly expressed in erythroid cells and megakaryocytes and is essential for their lineage differentiation [7,8,9]. Interactions of GATA1 with GFI1B, a major hematopoietic factor, as well as with the repressive MeCP1 complex and the chromatin remodeling ACF/WCRF complex, have been described [10]. GATA DNA-binding sites have been identified in several megakaryocyte-specific genes, including those encoding platelet receptors such as GPIbα, GPIbβ, GPIX, GPIIb, and GPVI [11,12,13,14]. GATA1 consists of a polypeptide chain containing a transactivation domain and two homologous zinc finger DNA-binding domains: the amino (N-) terminal finger (N-ZF) and the C-terminal zinc finger (C-ZF). The C-ZF is responsible for direct DNA-binding activity for most target genes [15], whereas the N-ZF typically interacts with the DNA via palindromic GATA1 motifs and with numerous transcriptional cofactors, including the most important coactivator friend-of-GATA1 (FOG1) [16], T-cell acute lymphocytic leukemia protein 1 (TAL1) [17], LIM domain only 2 (LMO2) [18], retinoblastoma protein (pRb) [19], and PU.1 [20]. Expression of the *GATA1* gene produces two splicing isoforms: a 413 amino acid long form, in which all six exons are translated, and a shorter spliced variant (sGATA1), in which exon 2 is omitted, lacking 83 amino acids. sGATA1 alone was shown incapable of fully supporting physiologic erythropoiesis [21]. Moreover, homozygous deletion of GATA1 caused embryonic lethality in mouse models because of major erythroid disruption [22]. Interestingly, individuals affected by Diamond–Blackfan anemia, a form of congenital erythroid aplasia, occasionally harbor GATA1 mutations located in close proximity to the exon 2 boundaries (c.220G>C, c.220delG and c.2T>C). Those patients appear to have either a splicing defect or a translational defect, which favors the production of sGATA1 and reduces levels of full-length GATA1 [23,24]. Moreover, acquired somatic mutations in GATA1 accompany the development of transient abnormal myelopoiesis and eventually acute megakaryoblastic leukemia associated with Down syndrome [25,26]. Being located on the X chromosome (p11.23), GATA1 germline variants are inherited in an X-linked pattern. Notably, different positions of the GATA1 pathogenic variants result in a remarkably high variety of phenotypes encompassing ineffective erythropoiesis, neutropenia, thrombocytopenia, and thrombocytopathy [27]. There is a solid body of evidence underlying the implications of GATA1 variants in erythropoietic disorders [24,28,29,30]. 

Over the past few decades, an increasing number of germline variants within the *GATA1* gene have been reported, which seem to lead to different phenotypic manifestations. For instance, p.V205M and p.G208R cause severe dyserythropoietic anemia and thrombocytopenia, p.R216Q leads to thrombocytopenia with beta-thalassemia, and p.V74L and splice-inducing variants in 5′UTR cause macrocytic or dyserythropoietic anemia and neutropenia, accompanied by either normal or increased platelet count [31,32,33,34,35,36]. A very rare GATA1 variant (p.*414Arg) enlarging GATA-1 is associated with the rare X-linked blood group Lu(a-b-) phenotype and mild macrothrombocytic thrombocytopenia [37]. In the Spanish project of IPD, we have recently identified a new pedigree with this p.*414Arg GATA1 variant (JR unpublished data). 

Interestingly, the majority of the reported mutations cluster within the N-terminal zinc finger domain in close proximity to each other. Previous studies demonstrated that different GATA1 variants affect GATA1–FOG1 binding to a variable extent. The GATA1 variants with weaker affinity to FOG1 appear to cause a more severe phenotype [8,36]. This might even apply to different amino acid exchanges at the same position, as described for the wild-type positions G208, R216, and D218. Indeed, two GATA1 variants have been reported with an amino acid exchange in position 208, namely G208R and G208S, resulting in severe to moderate macrothrombocytopenia with dyserythropoiesis, respectively with and without anemia. The substitution of a hydrophilic residue with a large positively charged amino acid, as in the p.G208R variant, was suggested to destabilize the GATA1–FOG1 interaction more profoundly than the exchange with the smaller hydrophilic serine, as for the p.G208S variant, and therefore was held responsible for the more severe phenotype [33]. Moreover, two GATA1 pathogenic variants leading to significantly different clinical constellations have been reported in position 216. Patients with the p.R216Q variant generally present moderate macrothrombocytopenia, α-granule deficiency, platelet dysfunction, and mild thalassemia. On the other hand, the exchange of arginine with tryptophane, instead of glutamine, as in the R216W GATA1 variant, has been described in a patient affected by anemia, splenomegaly, painful photosensitive bullous dermatosis, and hirsutism, a condition also known as congenital erythropoietic purpura [38]. Surprisingly, p.R216Q does not reduce affinity to FOG1, whereas it decreases the binding of GATA1 to specific palindromic DNA sequences [38,39]. The impact of the p.R216W variant on GATA1–FOG1 interaction is still unclear. This variant appears rather to affect the binding to cis-acting DNA elements, unveiling a further molecular mechanism possibly responsible for the remarkable relationship between genotype and phenotype [38]. Moreover, p.D218G has been described with mild macrothrombocytopenia with dyserythropoiesis [36], D218Y with severe macrothrombocytopenia with anemia [35], and D218N with macrothrombocytopenia with dyserythropoiesis [40]. 

In this article, we describe clinical cases of patients with GATA1 variants located in the two zinc fingers, characterized by impaired platelet function. Two unrelated male patients, one from Germany and one from Spain, share a novel variant located near the C-ZF domain leading to a platelet function defect but interestingly with mostly normal to borderline low platelet counts. We investigated the genotype/phenotype in these two patients and compared the findings with those in two German siblings (brothers) who carry the previously reported likely pathogenic variant p.D218N in the N-ZF domain and presented with thrombocytopenia.

## 2. Materials and Methods

### 2.1. Patients

Patients’ medical and bleeding histories were obtained by trained physicians from patient interviews and written medical reports. Hemorrhagic diathesis was quantified using the web-based version (bleedingscore.certe.nl (accessed on 20 April 2022) or standard form of the bleeding assessment tool of the International Society on Thrombosis and Haemostasis Scientific and Standardization Committee (ISTH-SSC BAT) [41,42].

Pedigree A: Two siblings, patients A I.1 and A I.2, were referred to the outpatient clinic in Freiburg, Germany, presenting with severe chronic thrombocytopenia (<50 × 10^9^/L). In patient A I.1, a low platelet count had been first noticed at the age of 4 years, as he was hospitalized because of a severe traumatic brain injury requiring surgical treatment. The surgery was performed under transfusion of platelet concentrates. Perioperatively, the boy did not experience any bleeding problems, and he developed normally afterward. He reported frequent hematomas lacking precedent adequate trauma, occasional epistaxis, and gingival bleeding. Physical examination showed multiple hematomas at different stages on the extremities and on the torso. His younger brother, patient A I.2, also suffered from hemorrhagic diathesis (atraumatic hematomas, epistaxis, and gingival bleeding). After tonsillectomy, patient A I.2 had developed postoperative bleeding despite prophylactic transfusion of platelet concentrates. After tooth extraction under transfusion of platelet concentrates, he did not develop any hemorrhagic complications. In patients A I.1 and A I.2, autoimmune etiologies of thrombocytopenia, as well as bone marrow failure, were ruled out before. Moreover, the mother (A II.1) of the two boys suffered from hypermenorrhoea and menorrhagia, occasionally leading to iron-deficiency anemia, which required oral iron substitution. However, her platelet count had always been within the lower normal range. The father (A II.2) was not affected. Laboratory findings for family A are summarized in Table 1. Interestingly, the mother of the two boys reported that her father and two uncles (A III.1, A III.2, and A III.3) suffered from thrombocytopenia of different degrees with diverse phenotypical manifestations ranging from frequent bruising to rare nose bleeding. Unfortunately, no medical record of them could be provided and their genetic status remains unclear. However, the family history seems to be compatible with the X-linked inheritance pattern.

Pedigree B: Patient B I.1 was referred to our Department of Paediatrics in Freiburg as a 13-year-old boy with prolonged bleeding and impaired wound healing following an operation on his toe. Moreover, in his childhood, he suffered from frequent nose bleeding, which occasionally lasted for several hours and could always be successfully treated conservatively. He also had recurrent atraumatic hematomas on his whole body. His sister (B I.2) reported no bleeding problems apart from menorrhagia. His mother (B II.1) also suffered from extensive menstrual bleeding. Furthermore, she recounted prolonged postnatal bleeding after both deliveries. The father (B II.2) displayed no bleeding history. Laboratory findings for family B are summarized in Table 2.

Pedigree C: Patient C I.1, currently a 43-year-old Spanish man, was first referred to our outpatient hematology clinic in Murcia as a 14-year-old boy with bleeding diathesis and suspected platelet dysfunction. Over years of follow-up, he has displayed common epistaxis, ecchymosis, disproportionate bruising, and hematomas with minor trauma and impaired wound healing. He received a platelet transfusion for inguinal hernia surgery. Bleeding after dental surgery required prophylaxis with tranexamic acid and desmopressin. His consanguineous parents and his sister and brother have no relevant bleeding history, but an aunt and a female cousin (not shown) reported frequent atraumatic hematomas, menorrhagia, and post-labor and surgical bleeding. Patient C I.1 fulfilled the criteria to be thereafter enrolled in the Spanish multicenter project “Functional and Molecular Characterization of Patients with Inherited Platelet Disorders” [43]. Laboratory findings for family C are summarized in Table 3.

### 2.2. Laboratory Test

#### 2.2.1. Cell Blood Count and Platelet Aggregometry Assays

Venous blood was drawn into either 7.5% K3 EDTA tubes (for hemogram and DNA purification) or 0.105–0.129M sodium citrate (for functional studies). Full blood count was measured using an automated cell counter (German pedigrees A and B with Sysmex KX-21 N, Norderstedt, Germany; Spanish pedigree C with Sysmex XS1000i, Sysmex España SL, Sant Just Desvern, Spain). Platelet-rich plasma (PRP) and platelet-poor plasma (PPP) were obtained by centrifugation of citrate-anticoagulated blood samples. 

For the German cases, platelet aggregometry was performed by using the APACT 4004 aggregometer (LABiTec, Ahrensburg, Germany), after stimulation of PRP with collagen (2 µg/mL; Takeda, Linz, Austria), adenosine diphosphate (ADP; 4 µmol/L; Sigma-Aldrich, St. Luis, MO, USA), epinephrine (8 µmol/L; Sanofi-Aventis, Frankfurt, Germany), and ristocetin (1.2 mg/mL; American Biochemical and Pharmaceutical LTD, Frankfurt, Germany).

Agonist-induced light transmission aggregometry in the Spanish pedigree was performed as described elsewhere [43], using an Aggrecorder II aggregometer (Menarini Diagnostics, Florence, Italy). Time course changes in the maximal percentage of light transmission of PRP over baseline PPP were recorded for 300 s upon stimulation with the specified platelet agonists and dose. PFA-100 tests (Siemens Healthcare, Barcelona, Spain) were performed following the manufacturer’s instructions.

#### 2.2.2. Flow Cytometry Assays

The flow cytometric assessment of platelets from the German patients was performed using FACSCalibur (Becton Dickinson, Heidelberg, Germany) [44]. Diluted PRP aliquots (5 × 10^7^/mL) were fixed and stained with FITC-labeled monoclonal surface antibody against glycoproteins (GPs) CD41 (GPIIb/IIIa-complex, integrin αIIbβ3), CD42a (GPIX), and CD42b (GPIb) (Coulter, Immunotech, Marseille, France). FITC-labeled anti-VWF (Bio-Rad AbD Serorech, Puchheim, Germany) and Alexa Fluor 488-labeled anti-fibrinogen (Invitrogen, Waltham, MA USA) were used to stain the platelets. In the presence of 1.25 mM Gly-Pro-Arg-Pro (Bachem, Bubendorf, Switzerland), diluted PRP (5 × 10^7^ platelets/mL) was stimulated with different concentrations of thrombin (0, 0.05, 0.1, 0.2, 0.5, and 1 U/mL; Siemens Healthineers, Marburg, Germany) to conduct the CD62 and CD63 expression analyses. Additionally, the platelets were stained with monoclonal FITC-labeled anti-CD62 (P-selectin) and anti-CD63 antibodies (lysosomal membrane-associated glycoprotein 3, LAMP-3; Immunotech, Marseille, France). Data of patients and controls (day control and 20 independent measurements from 10 controls as mean ± standard error of the mean (SEM)) were analyzed using GraphPad Prism software (version 8, San Diego, CA, USA). 

In the Spanish pedigree, the platelet expression of major membrane GPs, agonist-induced fibrinogen binding, and α- and δ-granule secretion (p-selectin or CD62 and CD63, respectively) were assessed by flow cytometry in an Accuri C6 flow cytometer (BD Biosciences), as detailed elsewhere [45]. Briefly, the expression of (GPIa (integrin α2), GPIbα, GPIX, GPIIb, GPIIIa (integrin αIIbβ3), and GPVI was evaluated by flow cytometry in citrated whole blood, diluted 1:10 in sterile phosphate-buffered saline (PBS), stained with specific antibodies (all from BD Biosciences, Madrid, Spain). To analyze platelet granule secretion and αIIbβ3 activation, diluted PRP (∼20 × 10^9^/L) was incubated (30 min, room temperature, static conditions) with Tyrode’s buffer, as control for non-stimulated platelets, or with the specified platelet agonists in the presence of anti-CD41*APC (as a platelet marker), fibrinogen-Alexa488 (Thermo Fisher Scientific, Waltham, MA, USA), and anti-CD62*PE (α-granule secretion) or anti-CD63*PE (dense granule secretion) (BD Biosciences, Madrid, Spain). Reactions were stopped with 4% paraformaldehyde (PFA) (*v*/*v*) (15 min, RT), and samples were diluted with PBS and run in the Accuri C6. For analysis, 10,000 platelets were gated on both CD41a positivity and forward scatter–side scatter (FSC-SSC), and results were expressed as the mean of median fluorescence intensity (MFI).

#### 2.2.3. Light and Immunofluorescence Microscopy

Platelet morphology in the Spanish index was assessed by light and immunofluorescence microscopy on blood smears as reported [46,47]. Particularly, the expression of three α-granule markers (i.e., thrombospondin, von Willebrand factor (vWF), and P-selectin), three dense granule markers (i.e., markers LAMP-1, LAMP-2, and CD63), and the cytoskeletal protein ß-tubulin was determined. The following primary antibodies were used: anti-thrombospondin (ab85762, Abcam, Cambridge, UK); anti-P-selectin (555522, BD Biosciences, San Jose, CA, USA); anti-vWF (A0082, Dako, Waldbronn, Germany); anti-LAMP-1 (sc18821, Santa Cruz Biotechnology, Heidelberg, Germany); anti-LAMP-2 (sc18822, Santa Cruz, CA, USA); anti-CD63 (558019, BD Biosciences); anti-β1-tubulin (T4026, Merck Life Science, Darmstadt, Germany). As secondary antibodies, Alexa Fluor 568 (A11011, Invitrogen, Thermo Fisher Scientific) and Alexa Fluor 488 (A11001, Invitrogen, Thermo Fisher Scientific, Dreieich, Germany) were used. Each marker was eventually assessed by standard immunofluorescence microscopy using the Olympus BX40 microscope system (Olympus, Hamburg, Germany). Blood smears from healthy controls were stained and analyzed in parallel. 

#### 2.2.4. Transmission Electron Microscopy Assays

Electron microscopy was used to examine platelet morphology and cytoskeletal network in the Spanish index case, as previously reported [48]. The obtained platelet sections were observed using a Philips/FEITecnai12 transmission electron microscope (FEI; Hillsboro, OR, USA) at 80 kV.

#### 2.2.5. Molecular Genetic Analyses

To extract genomic DNA from EDTA blood, we used standard procedures and the DNeasy blood and tissue kit produced by Qiagen (Qiagen GmbH, Hilden, Germany), and we quantified the results using a Qubit 2.0 fluorometer (ThermoFisher Scientific, Scientific, Waltham, MA, USA). DNAs from the German index patients (A I.1, A I.2, and B I.1) were analyzed by high-throughput sequencing (HTS) of a 95-gene panel using a custom-designed Nextera Rapid Enrichment Kit (Illumina, Inc., San Diego, CA, USA) previously described [49], followed by sequencing on a MiSeq (Illumina). For bioinformatics analysis, Sequence Pilot (JSI medical systems) and Alamut Visual Plus (SOPHiA GENETICS, Rolle, Switzerland) were used. Confirmation of the variants identified and segregation analysis were performed using direct sequencing. For the Spanish patient, DNA analysis was carried out by using the HTS gene panel previously described using an Illumina platform (Illumina, San Diego, CA, USA) [45,50]. Variant calling and annotation were performed using an in-house pipeline, based on VarScan v2.3.9, SAMTools v1.3.1, ANNOVAR, Ensembl-VEP v99, and dbNFSP v4.0a bioinformatic tools. General variant information was obtained using the Varsome tool (https://varsome.com (accessed on 1 July 2022) [51]. In either case, we followed the guidelines of the American College of Medical Genetics and Genomics and the Association for Molecular Pathology (ACMG/AMP) to assess the pathogenicity of the candidate variant [52]. The variants identified in the index cases by HTS were confirmed and segregated in the pedigrees by direct Sanger sequencing using specific primers, using an ABI 3130 automated sequencer. 

## 3. Results

### 3.1. Blood Count, Blood Smear, Coagulation Parameters, and Bleeding Score

At the time of initial presentation in our outpatient clinic, both patient A I.1 and patient A I.2 showed severe thrombocytopenia (defined as platelet count < 50 × 10^9^/L) without other hematological abnormalities (Table 1).

On the contrary, patient B I.1 displayed a normal platelet count; however, the peripheral blood smear showed anisocytic thrombocytes. His hemoglobin was within the lower normal range, and his erythrocytes appeared macrocytic and normochromic (Table 2). Interestingly, we observed a mild increase in the mean corpuscular volume over the time of seven years ranging from 95 and 97 to 101.5 fL.

The three patients from Germany (A I.1, A I.2, and B I.1) showed a prolonged in vivo bleeding time (Ivy) of >15 min, 14.5 min, and 15 min, respectively (normal value < 6 min). INR, activated partial thromboplastin time, fibrinogen, factor VIII activity, and von Willebrand parameters were within the normal range.

During 30 years of follow-up, the Spanish patient CI.1 has displayed platelet count ranging from normal values to mild thrombocytopenia (123–215 × 10^9^/L). Platelet volume as assessed in the automated counter has been within normal values, but anisocytic thrombocytes have been a common finding in his blood smears. No other relevant anomalies have been observed in his sequential hemograms. Coagulation studies have been normal (not shown). His PFA-100 closure time for collagen–epinephrine was severely prolonged. The patient has an ISTH-BAT score of 8. Blood parameters were normal in the patient’s parents and brothers (Table 3). 

### 3.2. Light Transmission Aggregometry Indicated a Platelet Functional Defect

**Figure 1:**
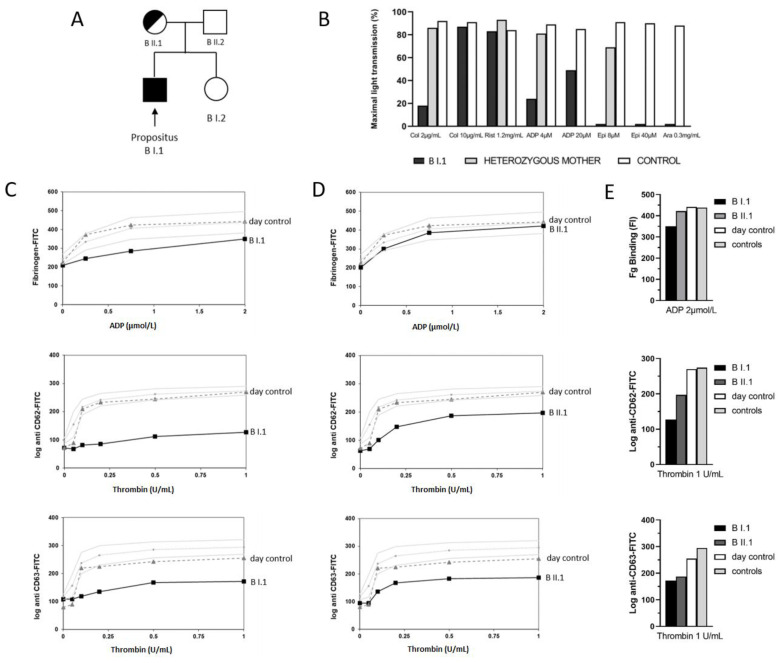
Family tree and platelet phenotyping in the German pedigree B. (**A**) Genetic pedigree of family B. Solid black symbols represent affected family member carrying the hemizygous GATA1 p.H289Y variant; black–white circles represent heterozygous female carrier of the variant. (**B**) LTA for propositus (B I.1) and his heterozygous mother (B II.1): low-dose collagen, arachidonic acid, and epinephrine failed to induce platelet aggregation, whereas platelet aggregation was reduced in response to ADP in B I.1. The heterozygous mother showed mildly reduced aggregation after stimulation with epinephrine. Ristocetin-induced agglutination was normal for both patients. Flow cytometry for propositus (**C**) and for the mother (**D**) as line diagram: only hemizygous propositus displayed reduced fibrinogen binding (**upper** panel); propositus and his mother showed reduced CD62 expression, more severe in the propositus (B I.1) (**middle** panel), and reduced CD63 expression (**lower** panel). Data of patients and controls (day control and 20 independent measurements from 10 controls as mean ± standard error of the mean (SEM)). (**E**) Combined histogram: bars correspond to fluorescence intensity (FI) values (log) at the highest concentration of the used stimulant.

**Figure 2:**
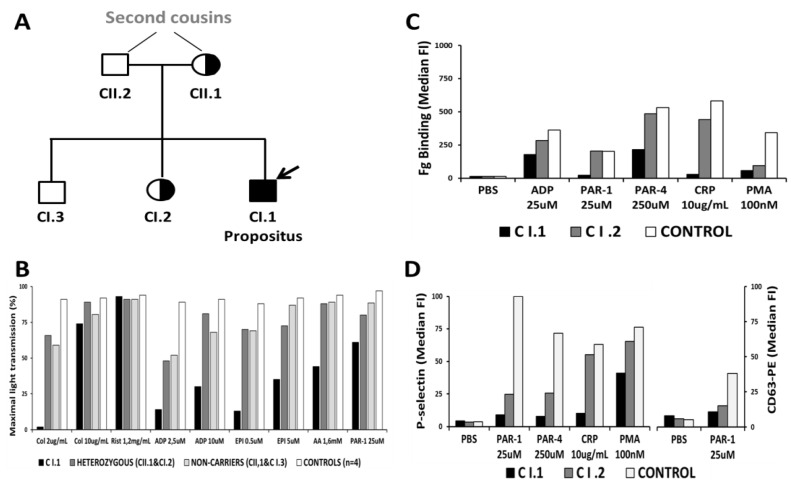
Family tree and platelet phenotyping of the Spanish pedigree C. In panel (**A**), black filled and half-filled symbols indicate hemizygosity and heterozygosity for the GATA1 p.H289Y variant, respectively. Panel (**B**) shows representative maximal platelet aggregation response in the hemizygous patient and mean aggregation values in his heterozygous and non-carrier relatives and in unrelated controls A similar aggregation defect has been observed several times in the patient over 30 years of follow-up. Panels (**C**,**D**) display agonist-induced αIIbβ3 and α- (P-selectin or CD62) and δ-granule (CD63) secretion in platelets from the patient, his healthy heterozygous sister, and a healthy unrelated control. Bars correspond to median fluorescence intensity (FI) values.

Light transmission aggregometry (LTA) performed for patients B I.1 (Figure 1B) and C I.1 (Figure 2B) showed severely impaired aggregation in response to multiple agonists, including collagen, ADP, epinephrin, arachidonic acid, and PAR-1, especially at a low agonist dose. In contrast, the ristocetin-induced platelet aggregation was unaffected. LTA was not performed for patients A I.1 and A I.2. because of the low platelet count (<50 × 10^9^/L). 

### 3.3. Platelet Flow Cytometric Analyses Indicated an α-Granule Defect

Platelet flow cytometric analysis of the patients in the three pedigrees (A I.1, A I.2, B I.1, and C I.1) demonstrated an expression of the major platelet receptors GPIb/IX, integrin αIIbβ3, GP Ia, and for the Spanish patient GPVI, comparable to parallel controls and within the normal range in healthy subjects.

**Figure 3:**
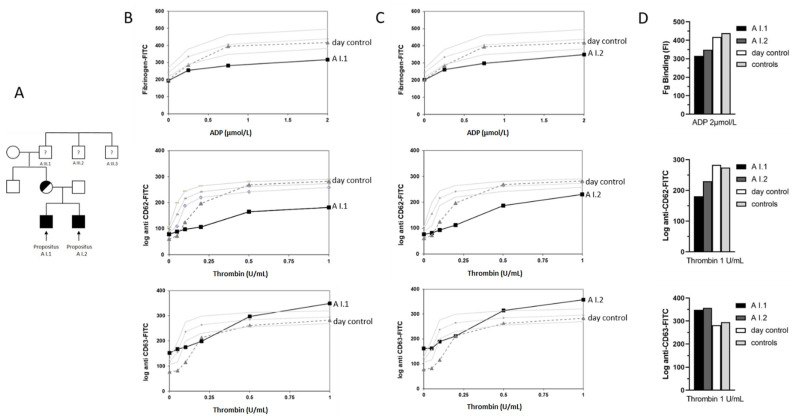
Family tree and platelet phenotyping in the German pedigree A. (**A**) Genetic pedigree of family A. Solid black symbols represent affected family members carrying the hemizygous GATA1 p.D218N variant; black–white circles represent heterozygous female carriers of the variant. Question mark represents male family members with reported phenotype without clinical or genetic data available. Flow cytometry for A I.1 (**B**) and for his younger brother A I.2 (**C**): both patients showed decreased fibrinogen binding (upper panel), severely reduced CD62 expression (middle panel), and slightly higher CD63 expression values after stimulation with high concentration of thrombin (lower panel) compared to healthy day control/controls (20 independent measurements from 10 controls as mean ± standard error of the mean (SEM)). (**D**) Bars correspond to fluorescence intensity (FI) values (log) at the highest concentration of the used stimulant.

In response to ADP and thrombin stimulation, platelets from patients A I.1 and A I.2 displayed a decreased fibrinogen binding and a reduced expression of CD62, hinting at an impaired agonist-induced αIIbβ3 activation and α-granule secretion. In contrast, the platelet release of CD63 after stimulation with increasing concentrations of thrombin reached slightly higher levels than that in the controls (Figure 3B and 3C for A I.1 and A I.2, respectively).

This pattern of agonist-induced platelet activation and granule secretion was similar in patient B I.1 and his mother (Figure 1). They showed reduced thrombin-induced α-granule secretion (Figure 1C,D middle panel). Only the hemizygous patient displayed reduced fibrinogen binding. The functional defect was clearly more marked in the patient than in his heterozygous mother. In contrast with findings in the siblings from pedigree A, platelets from patient B I.1 and his mother also displayed a moderately reduced agonist-induced CD63 expression, hinting at impaired δ-granule secretion.

Lastly, the Spanish patient C I.1 also displayed a markedly impaired agonist-induced fibrinogen binding and α- and δ-granule secretion in comparison to control platelets. His heterozygous sister showed a less pronounced reduction in these markers of platelet activation in response to some agonists (Figure 2C,D).

### 3.4. Platelet Immunofluorescence Microscopy, Morphology, and Ultrastructure for the Spanish Index Patient

**Figure 4:**
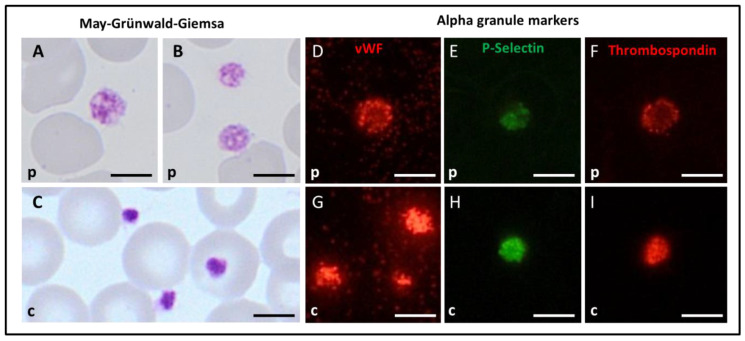
(**A**–**C**) In light microscopy, the patient’s platelets displayed increased size and reduced granularity compared to control. In addition, vacuoles were sometimes evident, particularly in the larger platelets. (**D**–**I**). In immunofluorescence microscopy, a mild reduction in the expression of the alpha granule markers von Willebrand factor, vWF (**D**), P-selectin (**E**), and thrombospondin (**F**) was noticeable in comparison to controls (**G**–**I**). Legend. p = C I.1; c = healthy control. Scale bars correspond to 5 µm.

Using light microscopy (Figure 4A–C), a heterogeneous platelet population with macrothrombocytes, in the absence of giant platelets, was found. A subpopulation of about 30–40% of the platelets, including the larger ones, displayed reduced granularity and sometimes displayed vacuoles. Using immunofluorescence microscopy (Figure 4D–I), a reduced expression of the markers for alpha granules was observed compared to controls. The other investigated markers were normally expressed.

**Figure 5:**
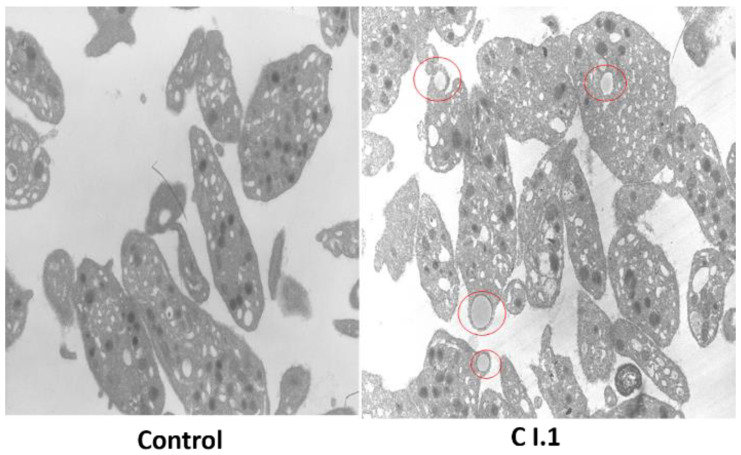
Platelet electron microscopy for the Spanish patient. Platelet electron microscopy could be performed once in the Spanish index case. Similar, or slightly reduced, numbers of alpha and dense granules are seen in the patient vs. control. C I.1′s platelets also show larger vacuole-type structures (red circles) which contain material, so they are not likely a part of the open canalicular system.

Platelet electron microscopy could be performed once in the Spanish index case. Alpha and dense granules were seen in patient platelets in similar, or slightly reduced for α-granules, numbers compared to those in control platelets. In this single analysis, C I.1 platelets also show larger vacuole-type structures containing material, so these are not likely a part of the open canalicular system (OCS) (Figure 5).

### 3.5. Molecular Genetic Analyses Identified GATA1 Variants in the Two Zinc Finger Domains

High-throughput sequencing revealed a hemizygous likely pathogenic variant (c.652G>A) in exon 4 of the *GATA1* gene (NM_002049.4) in patients A I.1 and A I.2 leading to an exchange of the amino acid aspartate in position 218 with asparagine (Asp218Asn, D218N) in GATA1 (Figure S1A). A swab DNA test in patients A I.1 and A I.2 confirmed the presence of the variant as a germline mutation. Family genotyping using direct sequencing revealed that the mother (A II.1) of the two brothers is a heterozygous carrier of the c.652G>A variant; the father presented with a wild-type sequence (Figure 3A). This variant was not reported in the gnomAD v2.1.1 database; however, it was listed in HGMD, as D218N has previously been reported [40]. In addition, variants with other amino acid exchanges in position D218 had previously been published: D218G [36] and D218Y [35]. In ClinVar (Accession No. RCV00085178.1), the variant is listed as likely pathogenic with macrothrombocytopenia. 

In patient B I.1 and independently in patient C I.1 from Spain, HTS analyses identified a novel hemizygous variant (c.865C>T) in exon 5 of the *GATA1* gene leading to an exchange of histidine with tyrosine in position 289 (His289Tyr, H289Y). Family genotyping identified B II.1 (mother) as a heterozygous carrier for the His289Tyr GATA1 variant. The father and sister of the patient presented with wild-type sequences (Figure 1A). The Spanish family genotyping revealed that the mother and sister of the male patient were heterozygous carriers of the variant (Figure 2A and Figure S1C). The H289Y variant is not yet reported in public population databases such as gnomAD (v2.1.1) and dbSNP (v151). Wild-type nucleotides and amino acids (down to nematode Caenorhabditis elegans) are highly conserved. Multiple lines of computational evidence support a deleterious effect on the encoded protein (SIFT, PolyPhen2). The Combined Annotation Dependent Depletion (CADD) score is 27.2—a CADD score greater than or equal to 20 indicates the 1% most deleterious substitutions. According to the currently available data on the variant and applying the ACMG standards and guidelines, we can classify the H289Y change as a variant of uncertain significance (VUS) (criteria PM2 moderate and PP3 supporting). Direct sequencing chromatograms for hemizygous and heterozygous variant carriers are displayed in the Supplementary Materials (Figure S1). In both patients, B I.1 and C I.1, no other rare non-synonymous coding variant (nsSNV) of uncertain significance could be identified in the genes investigated. 

## 4. Discussion

In this study, we examined three pedigrees with thrombocytopenia and platelet dysfunction associated with genetic variants in the *GATA1* gene (Supplemental Figure S2). 

The novelty is the identification of a new GATA1 variant in the C-terminal ZF of GATA1 described in unrelated German (pedigree B) and Spanish patients (pedigree C), independently. The C-ZF domain has been shown to be essential to induce megakaryocytic differentiation [53,54]. The hemizygous males (B I.1 and C I.1) presented with mucocutaneous bleeding and a platelet function disorder but without obvious thrombocytopenia, although patient C I.1 showed variable platelet counts ranging from 123 to 215 × 10^9^/L throughout the years. Full blood count showed mild anemia, anisocytosis, and poikilocytosis in both patients. In the genetic alteration (p.H289Y) identified in these patients, the basic amino acid histidine is replaced with tyrosine, a hydrophobic polar uncharged amino acid. Being located at the boundary of the C-terminal zinc finger domain, the exchange of a neutral residue (histidine) with a larger partially hydrophobic amino acid (tyrosine) could lead to conformational changes of the protein. Pereira et al. identified three hemizygous carriers of GATA1 p.H289R with mildly decreased or normal platelet counts (129, 208, and 185 × 10^9^/L, respectively) and mild macrocytic anemia when investigating a family with combined PKLR and GATA1 defect [30]. For these carriers of the GATA1 variant, no functional platelet analysis was performed. 

However, the predominant trait of the p.H289Y variant appears to be a qualitative defect of the platelets. Indeed, platelet LTA was impaired, and fibrinogen binding and CD62 and CD63 expression after stimulation were reduced in the hemizygous carriers B I.1 and C I.1. Mild to moderate hypogranulation of platelets, mainly the larger ones, was observed in blood smears of both patients. Light and immunofluorescence microscopy performed for C I.1 confirmed reduced granularity and a reduced expression of the markers for alpha granules compared to control. Electron microscopy analysis of platelets derived from patient C I.1 showed only a mild paucity of α-granules and large vacuoles, which were noticed in light microscopy as well. Regarding the reduction in CD62 (P-selectin) and CD63 expression (observed in flow cytometry), this may be related to an impaired platelet activation response to agonists rather than to a significant quantitative defect in granules. 

More recently, another novel GATA1 variant within the C-terminal zinc finger domain, p.Leu268Met (L268M), has been described in two brothers who suffered from prolonged bleeding and pronounced mucocutaneous hemorrhages. These patients showed severe platelet dysfunction and displayed a significant reduction in α- and δ-granules [53]. Postoperative bleeding after minor surgeries was prevented by the administration of platelet concentrates. A most remarkable observation was that the platelet count of both brothers, initially within the normal range, gradually decreased over time, causing mild to moderate thrombocytopenia. Both patients displayed anisocytosis and poikilocytosis as well as a progressive increase in platelet MCV over the years; however, they were never anemic. 

These are the first reported GATA1 variants that are located in the C-terminal zinc finger region and are associated with bleeding symptoms, platelet dysfunction, and only borderline/mild thrombocytopenia. Furthermore, patient B I.1′s hemoglobin value was within the lower normal range and erythrocytes were macrocytic, suggesting an implication of the p.H289Y variant in erythropoiesis. Similarly, patients with the p.L268M variant show no anemia but show progressively enlarged erythrocytes, possibly prodromal of a bone marrow failure [53]. Further studies and follow-up of patients are needed to monitor the impact of C-ZF variants on megakaryopoiesis and the red cell line. 

Additionally, we identified two brothers carrying the likely pathogenic p.D218N variant, located within the N-terminal zinc finger domain of GATA1. They presented with lifelong thrombocytopenia (<50 × 10^9^/L) and decreased expression of CD62 after stimulation with thrombin, hinting at an impaired platelet α-granule secretion (flow cytometry). D218N has already been reported by Hermans et al. in two related patients. Both suffered from spontaneous bleeding (epistaxis, hematomas, and gingival bleeding) since childhood and had splenomegaly for which they received surgical treatment. Moreover, both presented with moderate (68–71 × 10^9^/L) to severe (19–36 × 10^9^/L) macrothrombocytopenia (MPV 12.6–12.8 fL, normal range 7.5–9.2 fL). Both cousins showed mild features of dyserythropoiesis, including poikilocytosis, anisocytosis, and schizocytes, and Howell–Jolly bodies in erythrocytes, but no anemia [40]. Similarly, the two brothers described in this study did not show any signs of anemia. Pathogenic variants in the residue p.218 have been described before, with different severity for the phenotype of the patients: macrothrombocytopenia with dyserythropoiesis (p.D218G) [36] and severe macrothrombocytopenia with anemia (p.D218Y) [35]. It has been shown that p.D218G partially disrupts the interaction with FOG1 [36], whereas p.D218Y has a stronger loss of affinity for FOG1 and disturbs GATA1 self-association [35]. FOG1 contributes to the stability of DNA binding to a palindromic GATA recognition sequence [55]. Additionally, it has been shown that megakaryocytes from patients with the D218G or D218Y have an abnormal expression of the GATA1-regulated *NBEAL2* and *ITGB3* genes, more profoundly in D218Y. Surprisingly, immunoblot analyses showed absent NBEAL2 protein expression in GATA1-deficient platelets, and a DNA-binding assay confirmed the binding of GATA1 to NBEAL2 long-distance enhancer, explaining the paucity of α-granules in GATA1 deficiency [56]. Table 4 offers an overview of the diversity of the reported GATA1 variants located in the two zinc-finger domains including the DNA-binding sites and the C-terminal region. 

In summary, we identified a novel variant (c.865C>T; p.H289Y) in the GATA1 C-ZF region in two unrelated patients leading to bleeding symptoms and impaired platelet function. Interestingly, these patients presented with normal platelet counts or borderline low platelet counts. On the contrary, we present two brothers with the p.D218N variant in the GATA1 N-ZF domain who suffered from bleeding symptoms and thrombocytopenia. Our findings are in line with the data from Saultier et al. who described a patient carrying the C-ZF variant p.L268M with bleeding and severe platelet aggregation defects without early-onset thrombocytopenia. N206I localized in the N-ZF was associated, on the other hand, with severe thrombocytopenia (15 × 10^9^/L) in early life [54]. 

GATA1 variants in the C-ZF region could lead predominantly to a functional platelet defect and may be overlooked if platelet function (especially granule secretion and fibrinogen binding) has not been investigated. Long-term follow-up of the patients should be carried out in order to detect a possible change in erythropoiesis.

## Figures and Tables

**Table 1:** Pedigree A, laboratory values.

Individuals	GATA1 Status D218N	PLTs (×10^9^/L)	MPV (fL) (7–12)	RBCs (×10^12^/L)	Hb (g/dL)	MCV (fL)	WBCs (×10^9^/L)	Bleeding Time (Ivy) (2–6 min)	ISTH-SCC BAT Score (M < 4; F < 6)
A I.1 Propositus	Hemizygous	37 (153–345)	11.1	5.12 (4.38–5.92)	15 (12.9–17.7)	85.7 (78–95)	7.2 (3.86–11.2)	>15	10 *
A I.2 Propositus	Hemizygous	32 (148–358)	n.a.	5.47 (4.31–5.86)	13 (12.4–17.2)	74.2 (77–93)	5.7 (3.8–11.24)	14.5	11 *
A II.1 Mother	Heterozygous	151 (176–391)	10.1	4.9 (4–5.2)	13.2 (11.6–15.5)	80.3 (80–95)	6.1 (4–10.4)	4.5	1 *
A II.2 Father	Wild type	212 (146–328)	11.1	4.7 (4.5–5.8)	15.4 (13.5–17.6)	93 (80–95.5)	8.4 (3.9–9.8)	3.0	0

Age- and gender-specific normal values in round spaces. Hb = hemoglobin. MCV = mean corpuscular volume. MPV = mean platelet volume. n.a. = not available. PLTs = platelets. RBCs = red blood cells. WBCs = white blood cells. ISTH-SCC BAT = International Society on Thrombosis and Haemostasis Scientific and Standardization Committee Bleeding Assessment Tool. * A I.1: epistaxis, ecchymosis, oral cavity bleeding, surgery requiring transfusion, and CNS hemorrhage requiring intervention; A I.2: epistaxis, ecchymosis, oral cavity bleeding, prolonged bleeding after surgery requiring transfusion, tooth extraction requiring transfusion; A II.1: menorrhagia.

**Table 2:** Pedigree B, laboratory values.

Individuals	GATA1 Status H289Y	PLTs (×10^9^/L)	MPV (fL) (7–12)	RBCs (×10^12^/L)	Hb (g/dL)	MCV (fL)	WBCs (×10^9^/L)	Bleeding Time (Ivy) (2–6 min)	ISTH-SCC BAT Score (M < 4; F < 6)
B I.1 Propositus	Hemizygous	232 (146–328)	9.9	3.99 (4.5–5.8)	13.1 (13.5–17.6)	101.5	6.2 (3.9–9.8)	>15	10 *
B II.1 Mother	Heterozygous	251 (176–391)	9.0	4.35 (4–5.2)	13.5 (11.6–15.5)	91.7	7.2 (4–10.4)	4.5	2 *
B II.2 Father	Wildtype	239 (146–328)	11.0	5.25 (4.5–5.8)	15.2 (13.5–17.6)	83.2	5.2 (3.9–9.8)	n.a	0
B I.2 Sister	Wildtype	199 (176–391)	10.2	4.23 (4–5.2)	13.3 (11.6–15.5)	94.1	5.0 (4–10.4)	6.5	1 *

Age- and gender-specific normal values in round spaces. Hb = hemoglobin. MCV = mean corpuscular volume. MPV = mean platelet volume. N = normal range. n.a. = not available. PLTs = platelets. RBCs = red blood cells. WBCs = white blood cells. ISTH-SCC BAT = International Society on Thrombosis and Haemostasis Scientific and Standardization Committee Bleeding Assessment Tool. * B I.1: epistaxis, ecchymosis, impaired wound healing, and prolonged bleeding after surgery or injury/minor wounds; B I.2: menorrhagia; B II.1: menorrhagia, postpartum hemorrhage.

**Table 3:** Pedigree C, laboratory values.

Individuals	GATA1 Status H289Y	PLTs (×10^9^/L)	MPV (fL)	RBCs (×10^12^/L)	Hb (g/dL)	MCV (fL)	WBCs (×10^9^/L)	PFA-100 Col-Epi (s)	ISTH-SCC BAT Score (M < 4; F < 6)
CI.1 Propositus Year 2008	Hemizygous	123	8.2	3.72	12.4	97.0	6.0	>300 s	8
Year 2022		182	11.8	4.26	13.5	103.1	12.1		
CI.2 Sister Year 2008	Heterozygous	186	8.4	4.13	13.5	94.0	8.2	141	0
Year 2022		230	10.1	4.44	13.4	94.1	11.8		
CI.3 Brother	Wildtype	209	8.8	4.75	14.4	84.5	8.9	85	0
C2.1 Father	Wildtype	149	9.2	4.56	14.0	89.9	5.5	nr	0
C2.2 Mother	Heterozygous	185	8.8	4.27	13.8	91.6	7.5	171	0

Values of hemograms performed in years 2008 and 2022 are displayed for the propositus and his healthy sister also carrying the p.H289Y variant. Hb = hemoglobin. MCV = mean corpuscular volume. MPV = mean platelet volume. n.r. = not performed. PLTs = platelets. RBCs = red blood cells. WBCs = white blood cells. ISTH-SCC BAT = International Society on Thrombosis and Haemostasis Scientific and Standardization Committee Bleeding Assessment Tool. Normal platelet count and MPV range in healthy subjects from our cohort (*n* = 107): platelets: 142–359 × 10^9^/L; MPV: 9–12.8 fL.

**Table 4:** Germline missense variants located in the zinc finger domains including the DNA-binding sites and the C-terminal region of GATA1 (NM_002049.4).

c.DNA	Amino Acid	dbSNP(rs); ClinVar	Phenotype	Additional Information	Disease	References
Variants in the N-ZF and DNA-binding domain						
613G>A	V205M, Val205Met	rs104894815; pathogenic	Severe TP and dyserythropoietic anemia; heterozygous female: mild chronic TP	FOG-1 interaction impaired, UniProt: severe impairment of ZFPM1 binding and erythroid differentiation in vitro	XLT	Nichols et al. [32]
617A>T	N206I, Asn206Ile	-	Severe TP without anemia, bone marrow: mild dyserythropoiesis	Disrupted MYH10 silencing during megakaryopoiesis	XLT	Saultier et al. [53]
622G>A	G208R, Gly208Arg	rs587776454; pathogenic	TP and dyserythropoietic anemia	Decreased FOG1 binding, reduced transcriptional activation and repression, reduced megakaryocyte maturation (Campbell et al., [17])	XLT	Del Vecchio et al. [33], further reports available
622_623 delinsTC	G208S, Gly208Ser	rs137852312; pathogenic	MTP and severe bleeding, hypogranular macrothrombocytes, but most them contained some α-granules	FOG-1 interaction impaired, partially disrupts the interaction with ZFPM1	XLT	Mehaffey et al. [34], further report available
646C>T	R216W, Arg216Trp	rs387907207; pathogenic	Congenital erythropoietic porphyria, TP and thalassemia	Alters affinity of GATA1 either for FOG-1 or with GATA recognition sites	CEP	Phillips et al. [38]
647G>A	R216Q, Arg216Gln	rs104894809; pathogenic	TP with thalassemia, absence or paucity of α-granules	Does not affect ZFPM1 binding; reduced affinity to palindromic GATA sites; supports erythroid maturation less efficiently than wild-type GATA1	XLTT	Yu et al. [39], further reports available
**652G>A**	**D218N,** **Asp218Asn**	**rs104894808;** **ranges from VUS to pathogenic**	**TP, α-granule deficit**		**XLT**	**Hermans et al. [40];** **this study**
652G>T	D218Y, Asp218Tyr	rs104894808; pathogenic	MTP and marked anemia	FOG-1 interaction impaired	XLT	Freson et al. [35]
653A>G	D218G, Asp218Gly	rs104894816; pathogenic	MTP and mild dyserythropoiesis without anemia	FOG-1 interaction impaired, partially disrupts the interaction with ZFPM1	XLT	Freson et al. [36]
Variants in the C-ZF and DNA-binding domain						
788C>T	T263M, Thr263Met	-	Mild anemia, neutrophilia, thrombocytopenia, megakaryocyte proliferation with mild myelofibrosis in female carriers			Svidnicki et al. [57]
802C>A	L268M		Normal platelet count at the beginning, then developing TP, TP with major δ-granule deficit, blood smear: red blood cell anisocytosis and poikilocytosis			Saultier et al. [53]
**865C>T**	**H289Y,** **His289Tyr**		**Two unrelated carriers: normal to variable platelet count (123 to 215 × 10^9^/L), α- and δ-granule deficit**			**This study**
866A>G	H289R, His289Arg		Three hemizygous carriers: moderate decreased or normal platelet counts (129, 208, and 185 × 10^9^/L, respectively) and mild macrocytic anemia			Pereira et al. [30]
920G>A	R307H, Arg307His	-	Severe fetal anemia with sustained mild MTP (121 × 10^3^/µL) and hyperchromic macrocytosis	Prevents Ser310 phosphorylation	HA	Hetzer et al. [58]
	R307C, Arg307Cys	rs1057518396; ranges from VUS to pathogenic	Hemolytic anemia, mild TP, dyserythropoietic anemia		HA	Ludwig et al. [59]
Variants in C-terminal region of GATA1						
1240T>C	Term414Argext*41	rs587776456; pathogenic	Mild TP, and X-linked form of Lu(a-b-) blood group phenotype		MXLT	Singleton et al. [37]

TP, thrombocytopenia; MTP, macrothrombocytopenia; XDAT, X-linked thrombocytopenia; XLTT, X-linked thrombocytopenia with thalassemia; CEP, congenital erythropoietic porphyria; HA, hemolytic anemia, MXLT, mild X-linked thrombocytopenia, GATA1 regions according to PeCan/St. Jude Cloud (https://pecan.stjude.cloud, accessed on 26 July 2022). Bold: variants discussed in this manuscript.

## Data Availability

Not applicable.

## Supplementary Materials

The following supporting information can be downloaded at: https://www.mdpi.com/article/10.3390/cells11203223/s1, Figure S1: Direct sequencing for verification of HTS found GATA1 variants; Figure S2: Functional domains of the GATA1 protein and localization of reported and current variants.
