# Supplementary material for: A Novel GATA1 Variant in the C-Terminal Zinc Finger Compared with the Platelet Phenotype of Patients with A Likely Pathogenic Variant in the N-Terminal Zinc Finger
Source: Cells. 2022 Oct 14;11(20):3223. doi: 10.3390/cells11203223 (PMC9600848; doi:10.3390/cells11203223)
Supplement: Supplementary file 1 [file cells-1861055-supplementary.pdf › cells-1861055-supplementary.pdf]

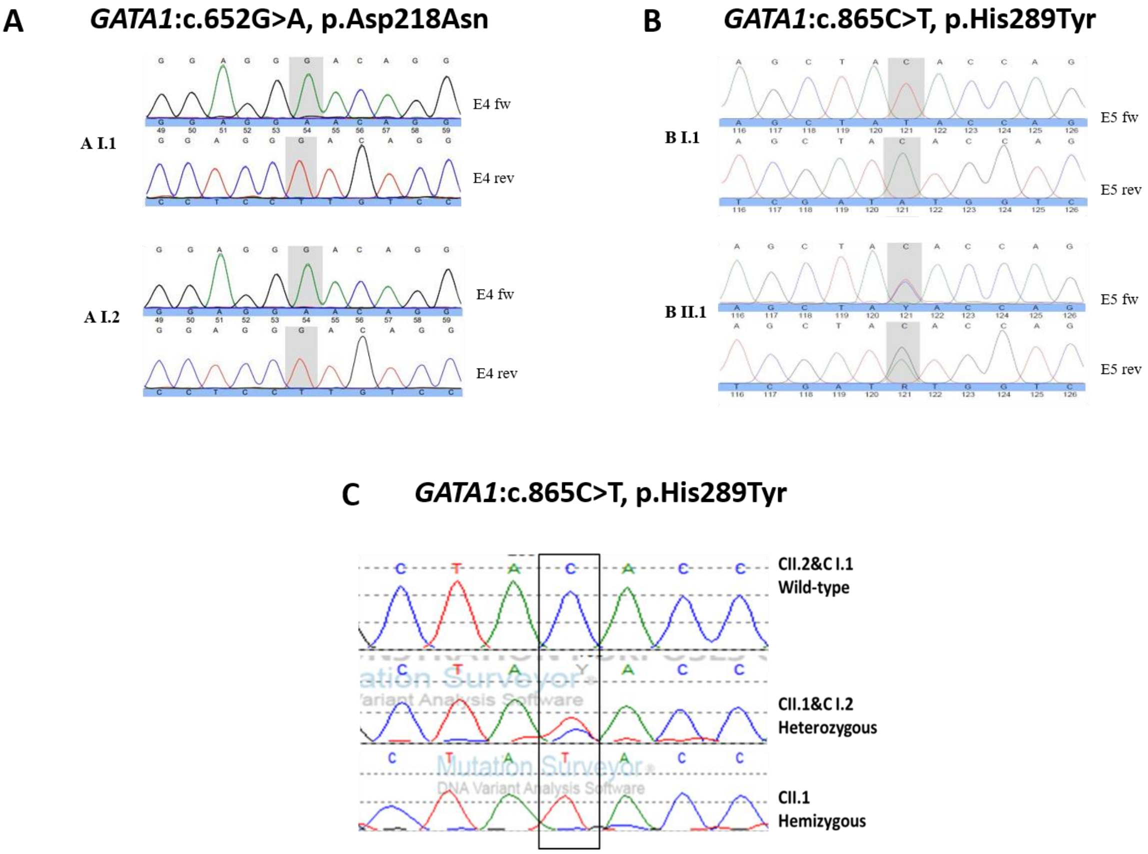

Figure S1. Direct sequencing for verification of HTS found GATA1 variants. (A) Hemizygous A I.1 and A I.2 carrying the GATA1 variant (marked in grey) c.652G>A leading to p.Asp218Asn. (B) Genetic analysis for hemizygous Propositus (B I.1) and his heterozygous mother B II.1 carrying the GATA1 variant c.865C>T, leading to His289Tyr. (C) Segregation by Sanger sequencing of the GATA1 variant c.865C>T in the Spanish pedigree.

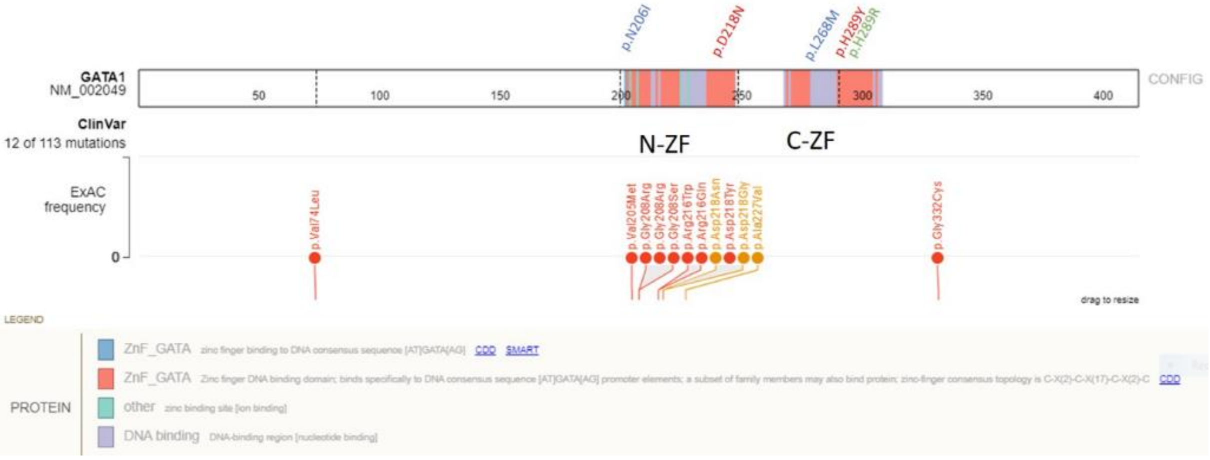

Figure S2. Functional domains of the GATA1 protein and localization of reported and current variants. Upper Panel: In red variants identified in this study, in blue variants reported by Saultier et al (2021), in green variant reported by Pereira et al (2016). Lower panel: Included are pathogenic (red) and likely pathogenic missense variants (orange) from ClinVar database. Modified graph provided by PeCan/St. Jude Cloud (https://pecan.stjude.cloud).
